# Effect of Oriented External Electric Fields on the Electronic Properties of Linear Acenes: A Thermally Assisted Occupation DFT Study

**DOI:** 10.3390/molecules29174245

**Published:** 2024-09-06

**Authors:** Chi-Yu Chen, Jeng-Da Chai

**Affiliations:** 1 Department of Physics, National Taiwan University, Taipei 10617, Taiwan; r09222069@ntu.edu.tw; 2 Center for Theoretical Physics and Center for Quantum Science and Engineering, National Taiwan University, Taipei 10617, Taiwan; 3 Physics Division, National Center for Theoretical Sciences, Taipei 10617, Taiwan

**Keywords:** TAO-DFT, OEEFs, electronic properties, acenes, multi-reference character

## Abstract

Recently, oriented external electric fields (OEEFs) have earned much attention due to the possibility of tuning the properties of electronic systems. From a theoretical perspective, one can resort to electronic structure calculations to understand how the direction and strength of OEEFs affect the properties of electronic systems. However, for multi-reference (MR) systems, calculations employing the popular Kohn–Sham density functional theory with the traditional semilocal and hybrid exchange–correlation energy functionals can yield erroneous results. Owing to its decent compromise between accuracy and efficiency for MR systems at the nanoscale (i.e., MR nanosystems), in this study, thermally assisted occupation density functional theory (TAO-DFT) is adopted to explore the electronic properties of *n*-acenes (*n* = 2–10), containing *n* linearly fused benzene rings, in OEEFs, where the OEEFs of various electric field strengths are applied along the long axes of *n*-acenes. According to our TAO-DFT calculations, the ground states of *n*-acenes in OEEFs are singlets for all the cases examined. The effect of OEEFs is shown to be significant on the vertical ionization potentials and vertical electron affinities of ground-state *n*-acenes with odd-number fused benzene rings. Moreover, the MR character of ground-state *n*-acenes in OEEFs increases with the increase in the acene length and/or the electric field strength.

## 1. Introduction

Among a wide variety of carbon materials, graphene (i.e., a two-dimensional system) has been of great interest due to its interesting properties and possible applications [1,2,3,4,5,6]. The long spin diffusion length and high carrier mobility of graphene offers attractive possibilities for graphene-based spintronics and electronics. Nevertheless, because graphene does not possess a band gap, it cannot be used for transistor applications.

To include a band gap into graphene, the carriers can be confined to quasi-one-dimensional systems, such as graphene nanoribbons (GNRs). Owing to their promising properties and potential applications, GNRs (i.e., narrow and long strips of graphene) have recently attracted a lot of attention from many researchers [7,8,9,10,11,12,13,14,15,16,17,18,19]. Because of the significant effects of edges and quantum confinement, the properties of GNRs are highly dependent on their edge shape (e.g., zigzag, armchair, or chiral) and geometrical structure (e.g., width and length). On the other hand, oriented external electric fields (OEEFs) [20,21,22,23,24,25,26,27,28,29,30,31] have also gained considerable attention due to the possibility of varying the properties of electronic systems in recent years. However, to date, there have been scarce studies on the effect of OEEFs on the properties of GNRs [26].

**Figure 1:**
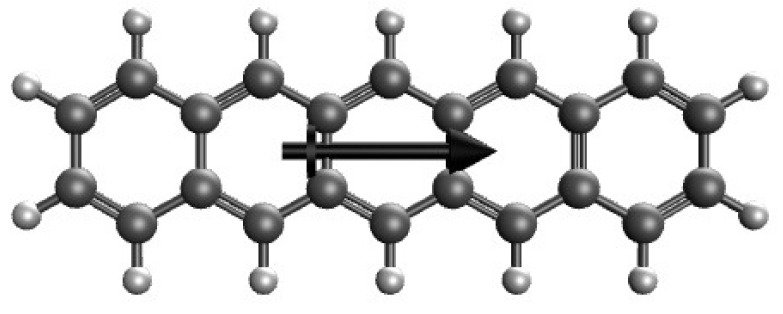
Structure of 5-acene, containing five linearly fused benzene rings, in an OEEF, where the arrow indicates the OEEF direction.

A good understanding of the properties of GNRs in OEEFs can benefit the relevant molecular design and potential applications. From a theoretical perspective, one can perform electronic structure calculations to know how the direction and strength of OEEFs affect the properties of GNRs. In this work, we conduct a computational study to investigate the OEEF effect on the electronic properties of the narrowest zigzag GNRs of various lengths. For finite-size models of the narrowest zigzag GNRs of various lengths, as illustrated in Figure 1, we take linear acenes (denoted as *n*-acenes, containing *n* linearly fused benzene rings), where the OEEFs of various electric field strengths *F* [given in atomic units (1 a.u. ≈ 51.4 V/Å)] are applied along the long axes of *n*-acenes. Accordingly, this study aims at exploring the electronic properties of *n*-acenes in OEEFs (with various values of *n* and *F*). While the electronic properties of *n*-acenes have been extensively studied [32,33,34,35,36,37,38,39,40,41], reports on the effect of OEEFs on the electronic properties of *n*-acenes are very scarce. Previous findings have shown that in the absence of OEEFs (i.e., F=0), the longer *n*-acenes have significant multi-reference (MR) character in their electronic ground states [32,33,34,35,36,37,38,39,40,41], suggesting that the longer *n*-acenes are electronic systems whose ground-state wave functions cannot be properly represented by single Slater determinants. Accordingly, it is expected that the longer *n*-acenes in OEEFs can also exhibit pronounced MR character in their electronic ground states.

Over the past thirty years, Kohn–Sham density functional theory (KS-DFT) [42,43] has been the most widely used electronic structure method for exploring the ground-state properties of electronic systems at the nanoscale (i.e., nanosystems), especially for systems with single-reference (SR) character in their electronic ground states (the so-called SR systems). Nonetheless, for systems with MR character in their electronic ground states (the so-called MR systems), calculations employing KS-DFT with the traditional semilocal [44,45,46] and hybrid [47,48,49] exchange–correlation (xc) energy functionals can yield erroneous results due to the presence of strong static correlation effects in MR systems [50,51,52,53]. On the other hand, to reliably predict the ground-state properties of small MR systems, one typically resorts to ab initio MR electronic structure methods [32,34,38,54,55,56,57,58,59,60]. However, for MR nanosystems, reliably accurate MR electronic structure methods are inapplicable as the computational cost of performing these MR calculations can be prohibitively high. Therefore, it is essential to adopt an efficient and reliable electronic structure method for studying the ground-state properties of MR nanosystems (e.g., the longer *n*-acenes).

Thermally assisted occupation density functional theory (TAO-DFT) [33] has recently emerged as a cost-effective solution to the challenge of studying MR nanosystems [36,37,39,40]. TAO-DFT, which has a similar computational cost as KS-DFT, is suitable for studying the ground-state properties of nanosystems. For an MR system, the representability of the ground-state electron density, which incorporates fractional orbital occupations generated by the Fermi–Dirac (FD) distribution function with some fictitious temperature θ, in TAO-DFT can be greatly improved [33,61], when compared with that in KS-DFT. Moreover, in TAO-DFT [33], the static correlation energy of an electronic system can be approximately described by the entropy contribution (absent in KS-DFT). The semilocal [33,36] and hybrid [39,62] exchange–correlation-θ (xcθ) energy functionals (i.e., the combined xc and θ-dependent energy functionals) can also be adopted in TAO-DFT. In addition, the popular dispersion correction schemes [63,64] can also be used in TAO-DFT for the efficient calculations of non-covalent interactions [36,39,65]. Simple schemes for determining the optimal system-independent [40] and system-dependent [66] θ values of an xcθ energy functional in TAO-DFT have been recently developed. Moreover, the fundamental distinction among three different electronic structure methods, such as finite-temperature density functional theory (FT-DFT) [43,67], TAO-DFT [33], and KS-DFT [42,43], has been discussed in a recent study [68].

Within the framework of TAO-DFT, a number of extensions, such as TAO-DFT with the polarizable continuum model (TAO-PCM) [69], TAO-DFT-based ab initio molecular dynamics (TAO-AIMD) [70], and a real-time extension of TAO-DFT (RT-TAO-DFT) [68], have been recently proposed for diverse applications. For example, TAO-PCM has been used to study the electronic properties of linear acenes in different solvents. In addition, TAO-AIMD simulations have been carried out to investigate the instantaneous/average MR character and infrared spectra of linear acenes at 300 K. In addition, RT-TAO-DFT calculations have been performed to explore the time-dependent properties (e.g., the number of bound electrons and high-order harmonic generation spectrum) of molecular hydrogen at the equilibrium and stretched geometries, subject to an intense laser pulse. Over the past few years, TAO-DFT and its extensions have been employed to explore a very wide range of properties (e.g., electronic [37,65,71,72,73,74,75,76], hydrogen storage [65], spectroscopic [70,77,78], and equilibrium thermodynamic [70] properties) of MR nanosystems.

Because TAO-DFT is a well-suited electronic structure method for studying MR nanosystems due to its decent compromise between accuracy and efficiency, in this study, we adopt TAO-DFT to obtain the electronic properties (e.g., singlet–triplet energy gaps, vertical ionization potentials, vertical electron affinities, fundamental gaps, symmetrized von Neumann entropy, active orbital occupation numbers, and real-space representation of active orbitals) of *n*-acenes (*n* = 2–10) in OEEFs of various electric field strengths *F*.

## 2. Computational Details

All the calculations are performed with Q-Chem 4.3 [79], using the 6-31G(d) basis set. The calculations are carried out using TAO-LDA (i.e., TAO-DFT with the local density approximation (LDA) xcθ energy functional) with the recommended fictitious temperature θ = 7 mhartree [33] to obtain the electronic properties of *n*-acenes (*n* = 2–10) in OEEFs, where the OEEFs of various electric field strengths *F* = 0.000, 0.001, 0.002, 0.003, 0.004, and 0.005 a.u. are applied along the long axes of *n*-acenes.

## 3. Results and Discussion

### 3.1. Singlet–Triplet Energy Gap

The singlet–triplet (ST) energy gap (the so-called ST gap), i.e., the energy splitting between the lowest triplet and singlet states, of a molecule offers an understanding of its ground-state nature, which could also be helpful to explore the possible MR character of the molecule [80,81,82,83,84] and to offer useful information for photovoltaic applications [85].

In this work, the ST gap ($E_{\mathrm{ST}}$) of *n*-acene in an OEEF is computed using

(1) $$E_{\mathrm{ST}}=E_{\mathrm{UT}}-E_{\mathrm{US}},$$

where $E_{\mathrm{UT}}$/$E_{\mathrm{US}}$ is the spin-unrestricted TAO-LDA energy of the lowest triplet/singlet state of *n*-acene in an OEEF (evaluated at the optimized lowest triplet/singlet-state molecular geometry).

**Figure 2:**
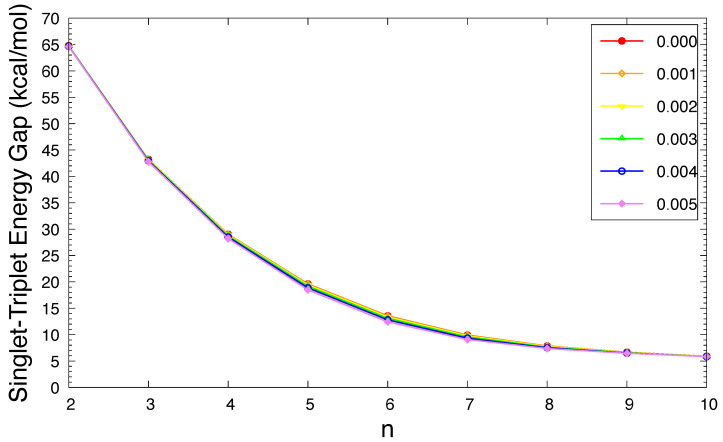
The singlet–triplet energy gap of *n*-acene in an OEEF of the electric field strength *F* = 0.000, 0.001, 0.002, 0.003, 0.004, and 0.005 a.u., calculated using spin-unrestricted TAO-LDA.

As shown in Figure 2, in an OEEF, the ST gap of *n*-acene decreases in a monotonic manner with an increasing acene length (also see Table S1 in Supplementary Information (SI)). As the electric field strength *F* increases, the ST gap of *n*-acene slightly decreases. For each case (i.e., *n* and *F*), the singlet state has lower energy than the triplet state. Therefore, for all the OEEFs considered, *n*-acenes (*n* = 2–10) possess singlet ground states. As MR systems generally have small ST gaps [32,33,34,36,37,38,39,40,71,72], the ground states of the longer *n*-acenes in OEEFs can have MR character.

On the other hand, the spin-symmetry constraint, which can be satisfied by an exact theory, ensures that the exact spin-unrestricted and spin-restricted calculations must yield the same energy for the lowest singlet state of an MR system. Nonetheless, such a spin-symmetry constraint can be greatly violated by KS-DFT with the traditional semilocal and hybrid xc energy functionals as well as SR electronic structure methods (e.g., the Hartree–Fock theory) [33,35,36,37,39,51,61,68,69,70], leading to strikingly different energies for the lowest singlet state of an MR system (the so-called spin-symmetry breaking). In principle, one can adopt reliably accurate MR electronic structure methods to resolve this problem. In practice, such MR methods are, however, very impractical for studying MR nanosystems (e.g., the longer *n*-acenes) because of the prohibitively high computational cost. Very recently, a TAO-DFT-based response theory [61] has been proposed to demonstrate that for any MR system, TAO-DFT with a sufficiently large fictitious temperature θ can always resolve the spin-symmetry breaking problem.

Here, we investigate whether this spin-symmetry constraint can be satisfied by TAO-LDA (i.e., with the recommended fictitious temperature θ = 7 mhartree) [33] and, hence, carry out additional spin-restricted TAO-LDA calculations for the lowest singlet energies of *n*-acenes in OEEFs (evaluated at the respective optimized molecular geometries). We find that within the numerical precision considered, the spin-restricted and spin-unrestricted TAO-LDA energies for the lowest singlet states of *n*-acenes in OEEFs are essentially identical [for each case (i.e., *n* and *F*), the energy difference is less than 0.001 kcal/mol], implying that, for all the cases examined, essentially no unphysical spin-symmetry breaking effects occur in our spin-unrestricted TAO-LDA solutions.

### 3.2. Vertical Ionization Potential, Vertical Electron Affinity, and Fundamental Gap

The vertical ionization potential, vertical electron affinity, and fundamental gap of a ground-state molecule are also key electronic properties. These properties are important in spectroscopy and catalyst selection and are also essential for choosing candidate materials for electronic devices and solar cells. Based on their definitions, the vertical ionization potential is the energy change when an electron is removed from the ground-state molecule (without altering the ground-state molecular geometry), the vertical electron affinity is the energy change when an electron is added to the ground-state molecule (without altering the ground-state molecular geometry), and the fundamental gap is the difference between the vertical ionization potential and vertical electron affinity.

At the spin-unrestricted TAO-LDA-optimized geometry of ground-state *n*-acene in an OEEF, we compute the vertical ionization potential [36,37,39]

(2) $${\mathrm{IP}}_{v}=E_{N-1}-E_{N},$$

vertical electron affinity

(3) $${\mathrm{EA}}_{v}=E_{N}-E_{N+1},$$

and fundamental gap

(4) $$E_{g}={\mathrm{IP}}_{v}-{\mathrm{EA}}_{v}$$

of ground-state *n*-acene in an OEEF. Here, $E_{N}$ is the total energy of the *N*-electron molecule (i.e., ground-state *n*-acene) in an OEEF, obtained with spin-unrestricted TAO-LDA.

**Figure 3:**
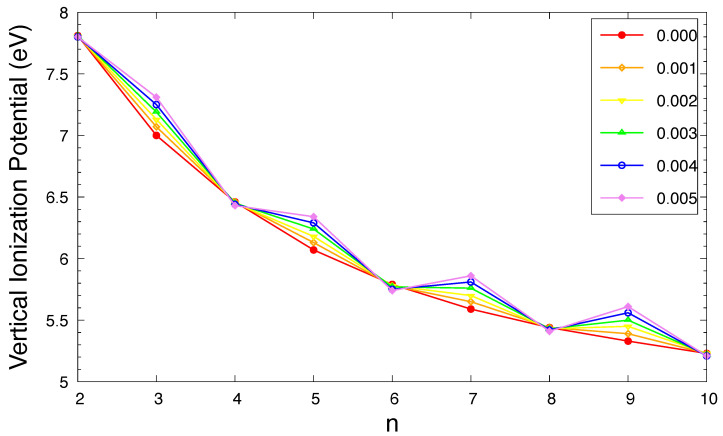
Vertical ionization potential for the ground state of *n*-acene in an OEEF of the electric field strength *F* = 0.000, 0.001, 0.002, 0.003, 0.004, and 0.005 a.u., calculated using spin-unrestricted TAO-LDA.

**Figure 4:**
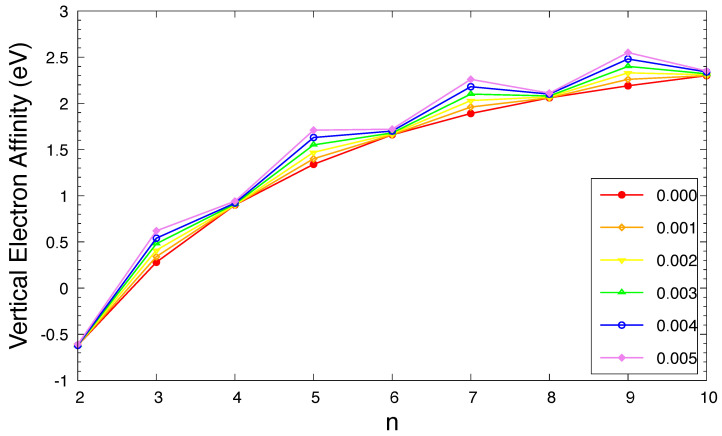
Vertical electron affinity for the ground state of *n*-acene in an OEEF of the electric field strength *F* = 0.000, 0.001, 0.002, 0.003, 0.004, and 0.005 a.u., calculated using spin-unrestricted TAO-LDA.

**Figure 5:**
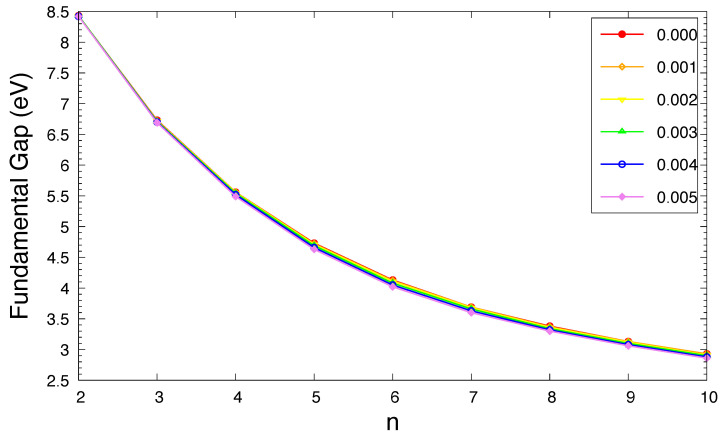
Fundamental gap for the ground state of *n*-acene in an OEEF of the electric field strength *F* = 0.000, 0.001, 0.002, 0.003, 0.004, and 0.005 a.u., calculated using spin-unrestricted TAO-LDA.

The ${\mathrm{IP}}_{v}$, ${\mathrm{EA}}_{v}$, and $E_{g}$ of ground-state *n*-acene in an OEEF are presented in Figure 3, Figure 4, and Figure 5, respectively (also see Tables S2–S4 in SI). For the smaller electric field strength *F* (e.g., F≤0.001 a.u.), with the increase in the acene length, the ${\mathrm{IP}}_{v}$ monotonically decreases, the ${\mathrm{EA}}_{v}$ monotonically increases, and hence the $E_{g}$ monotonically decreases, similar to those found in the absence of OEEFs (i.e., F=0) [36,37]. For the larger *F* (e.g., F≥0.002 a.u.), the ${\mathrm{IP}}_{v}$ and ${\mathrm{EA}}_{v}$ display odd–even oscillation patterns, while the $E_{g}$ shows a similar trend as that observed for the smaller *F* (e.g., F≤0.001 a.u.).

As *F* increases, both of the ${\mathrm{IP}}_{v}$ and ${\mathrm{EA}}_{v}$ greatly increase for *n*-acene with odd-number fused benzene rings, while the ${\mathrm{IP}}_{v}$/${\mathrm{EA}}_{v}$ slightly decreases/increases for *n*-acene with even-number fused benzene rings. As a consequence, the $E_{g}$ of ground-state *n*-acene slightly decreases with increasing *F*. In general, electronic systems with the fundamental gaps of 1–3 eV are suitable for photovoltaic applications. Therefore, among all the cases examined, 10-acene in OEEFs of *F* = 0.000, 0.001, 0.002, 0.003, 0.004, and 0.005 a.u. can be appropriate for photovoltaic applications.

### 3.3. Symmetrized von Neumann Entropy

We investigate the MR character of ground-state *n*-acene in an OEEF using the symmetrized von Neumann entropy [35,36,37,39]

(5) $$S_{\mathrm{vN}}=-\frac{1}{2}\sum_{\sigma =↑,↓}\sum_{i=1}^{\infty }\{f_{i,\sigma }\;\ln\left(f_{i,\sigma }\right)+\left(1-f_{i,\sigma }\right)\;\ln\left(1-f_{i,\sigma }\right)\}.$$

Here, σ = ↑ or ↓ denotes the up-spin or down-spin, and $f_{i,\sigma }$ (i.e., a value between 0 and 1) is the occupation number of the $i^{\mathrm{th}}$σ-spin orbital, obtained with spin-unrestricted TAO-LDA [33], approximately yielding the occupation number of the $i^{\mathrm{th}}$σ-spin natural orbital [71,86]. Based on Equation (5), the terms with $f_{i,\sigma }$ = 0 or 1 give no contribution to the $S_{\mathrm{vN}}$, while the terms with $f_{i,\sigma }$ greatly deviating from 0 and 1 (e.g., close to 0.5) can lead to a large increase in the $S_{\mathrm{vN}}$. Consequently, for an SR system, because all values of $f_{i,\sigma }$ are very close to 0 or 1, the $S_{\mathrm{vN}}$ should be vanishingly small. By contrast, for an MR system, because the occupation numbers of active f [i.e., those with noticeable fractional occupation numbers (e.g., between 0.1 and 0.9)] are closer to 0.5, and/or the number of active spin orbitals increases, the $S_{\mathrm{vN}}$ can be very large.

**Figure 6:**
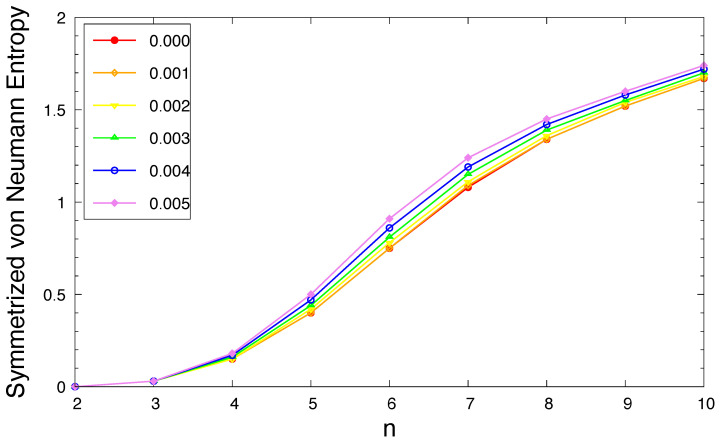
Symmetrized von Neumann entropy for the ground state of *n*-acene in an OEEF of the electric field strength *F* = 0.000, 0.001, 0.002, 0.003, 0.004, and 0.005 a.u., calculated using spin-unrestricted TAO-LDA.

As shown in Figure 6, the $S_{\mathrm{vN}}$ of ground-state *n*-acene in an OEEF monotonically increases with an increasing acene length (also see Table S5 in SI). This implies that the MR character of ground-state *n*-acene in an OEEF should generally increase with an increasing acene length. In addition, as the electric field strength *F* increases, the $S_{\mathrm{vN}}$ (i.e., a measure of MR character) of ground-state *n*-acene slightly increases.

### 3.4. Active Orbital Occupation Numbers

According to the aforementioned spin-unrestricted TAO-DFT calculations, with increasing acene length, the $S_{\mathrm{vN}}$ of ground-state *n*-acene in an OEEF monotonically increases. This indicates that as *n* increases, the occupation numbers of active spin orbitals are closer to 0.5, and/or the number of active spin orbitals increases. In spin-restricted TAO-DFT, this implies that for the ground state (i.e., lowest singlet state) of *n*-acene (containing *N* electrons) in an OEEF, as *n* increases, the occupation numbers of active orbitals [i.e., those with noticeable fractional occupation numbers (e.g., between 0.2 and 1.8)] are closer to 1, and/or the number of active orbitals increases. Here, the (N/2)th orbital is defined as the HOMO (highest occupied molecular orbital), and the (N/2+1)th orbital is defined as the LUMO (lowest unoccupied molecular orbital) [33,37,39].

**Figure 7:**
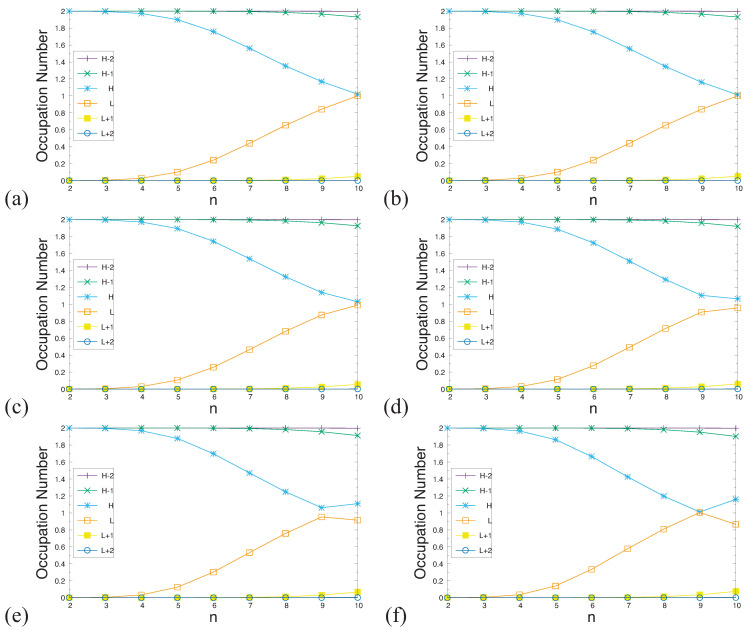
Occupation numbers of active orbitals for the ground state of *n*-acene in an OEEF of the electric field strength *F* = (**a**) 0.000, (**b**) 0.001, (**c**) 0.002, (**d**) 0.003, (**e**) 0.004, and (**f**) 0.005 a.u., calculated using spin-restricted TAO-LDA. Here, the HOMO/LUMO is denoted as the H/L for brevity.

To demonstrate this, in Figure 7, we present the occupation numbers of active orbitals for the ground state of *n*-acene in an OEEF, computed using spin-restricted TAO-LDA. As shown, the shorter *n*-acenes (e.g., n≤5) in an OEEF possess a nonradical nature in their ground states, because all the orbital occupation numbers are very close to 0 or 2. With the increase in the acene length, the occupation numbers of active orbitals are closer to 1, and/or the number of active orbitals increases, suggesting that the longer *n*-acenes in an OEEF possess an increasing polyradical nature in their ground states. Therefore, as the acene length increases, there is a transition from the nonradical nature of the shorter *n*-acenes to the increasing polyradical nature of the longer *n*-acenes in an OEEF. For *n*-acenes in an OEEF of the larger electric field strength *F*, the evolution of the polyradical nature is more rapid.

It is worth mentioning that with increasing acene length, the occupation numbers of active orbitals exhibit a curve-crossing behavior in the approach to 1. For example, in an OEEF, the orbital with the HOMO/LUMO character in the shorter *n*-acenes can become the LUMO/HOMO in the longer *n*-acenes. This curve-crossing behavior has been reported by the recent studies of *n*-acenes in the absence of OEEFs (i.e., F=0), using TAO-DFT [33,37,39,40] as well as an accurate MR electronic structure method [38]. This shows the reliability of TAO-DFT in the prediction of curve-crossing behavior.

### 3.5. Real-Space Representation of Active Orbitals

Here, we report the real-space representation of active orbitals (HOMO and LUMO) for the ground states of *n*-acenes (*n* = 2–10) in OEEFs, obtained with spin-restricted TAO-LDA (see Figures S1–S9 in SI). The edge localization of active orbitals for *n*-acenes in OEEFs is observed, similar to the edge localization of active orbitals previously found for *n*-acenes in the absence of OEEFs (i.e., F=0) [32,34,35,37].

As mentioned previously, the curve-crossing behavior is observed and can be easily seen here. For example, in an OEEF of the larger electric field strength *F* (e.g., F≥0.002 a.u.), the orbital with the HOMO/LUMO character in the shorter *n*-acenes (*n* = 2–9) becomes the LUMO/HOMO in 10-acene. Alternatively, it can also be regarded that for 10-acene in an OEEF, the curve-crossing behavior can occur when *F* is sufficiently large (e.g., F≥0.002 a.u.), highlighting the role of OEEFs.

## 4. Conclusions

In conclusion, we have employed TAO-DFT to explore the electronic properties of *n*-acenes (*n* = 2–10) in OEEFs, where the OEEFs of various electric field strengths *F* = 0.000, 0.001, 0.002, 0.003, 0.004, and 0.005 a.u. are applied along the long axes of *n*-acenes. Because the longer *n*-acenes (e.g., n≥6) in OEEFs have been shown to possess pronounced MR character in their ground states, KS-DFT with the traditional semilocal and hybrid xc energy functionals can yield incorrect results for these MR systems [33,36,37,39,40]. On the other hand, owing to their prohibitively high computational cost, reliably accurate MR electronic structure methods are generally inapplicable for exploring the electronic properties of MR nanosystems (e.g., the longer *n*-acenes) in OEEFs. Therefore, TAO-DFT seems to be a promising electronic structure method for the present study due to its decent compromise between accuracy and efficiency.

According to our TAO-DFT calculations, the ST gap of *n*-acene in an OEEF monotonically decreases with an increasing acene length. For all the OEEFs considered, *n*-acenes (*n* = 2–10) possess singlet ground states. The use of OEEFs has been shown to be significant for tuning the vertical ionization potentials and vertical electron affinities of ground-state *n*-acenes with odd-number fused benzene rings. For the smaller *F* (e.g., F≤0.001 a.u.), as *n* increases, the vertical ionization potential and fundamental gap monotonically decrease, while the vertical electron affinity and symmetrized von Neumann entropy monotonically increase, similar to those found in the absence of OEEFs (i.e., F=0) [36,37]. For the larger *F* (e.g., F≥0.002 a.u.), the vertical ionization potential and vertical electron affinity display odd–even oscillation patterns, while the fundamental gap and symmetrized von Neumann entropy show similar trends as those observed for the smaller *F* (e.g., F≤0.001 a.u.). The odd–even oscillation patterns of vertical ionization potential and vertical electron affinity are rather unexpected and deserve further investigations. Similar to the findings of previous studies on *n*-acenes in the absence of OEEFs (i.e., F=0) [35,37], the shorter *n*-acenes (e.g., n≤5) in an OEEF possess a nonradical nature in their ground states, and the longer *n*-acenes in an OEEF possess an increasing polyradical nature in their ground states. Therefore, with the increase in the acene length, there is a transition from the nonradical nature of the shorter *n*-acenes to the increasing polyradical nature of the longer *n*-acenes in an OEEF. For *n*-acenes in an OEEF of the larger *F*, the evolution of the polyradical nature is more rapid. For future work, other MR systems (e.g., the coronene series [72] and other alternant polycyclic aromatic hydrocarbons [71]) in the OEEFs of various directions and strengths may be studied.

## Data Availability

The data supporting this article have been included as part of the Supplementary Information.

## Supplementary Materials

The following supporting information can be downloaded at: https://www.mdpi.com/article/10.3390/molecules29174245/s1, Figure S1: Real-space representation of the HOMO (left) and LUMO (right) for the ground state of 2-acene in an OEEF of the electric field strength *F* = (a) 0.000, (b) 0.001, (c) 0.002, (d) 0.003, (e) 0.004, and (f) 0.005 a.u., calculated using spin-restricted TAO-LDA, at an isovalue of 0.02 e/Å^3^, where the orbital occupation numbers are shown in parentheses; Figure S2: Real-space representation of the HOMO (left) and LUMO (right) for the ground state of 3-acene in an OEEF of the electric field strength *F* = (a) 0.000, (b) 0.001, (c) 0.002, (d) 0.003, (e) 0.004, and (f) 0.005 a.u., calculated using spin-restricted TAO-LDA, at an isovalue of 0.02 e/Å^3^, where the orbital occupation numbers are shown in parentheses; Figure S3: Real-space representation of the HOMO (left) and LUMO (right) for the ground state of 4-acene in an OEEF of the electric field strength *F* = (a) 0.000, (b) 0.001, (c) 0.002, (d) 0.003, (e) 0.004, and (f) 0.005 a.u., calculated using spin-restricted TAO-LDA, at an isovalue of 0.02 e/Å^3^, where the orbital occupation numbers are shown in parentheses; Figure S4: Real-space representation of the HOMO (left) and LUMO (right) for the ground state of 5-acene in an OEEF of the electric field strength *F* = (a) 0.000, (b) 0.001, (c) 0.002, (d) 0.003, (e) 0.004, and (f) 0.005 a.u., calculated using spin-restricted TAO-LDA, at an isovalue of 0.02 e/Å^3^, where the orbital occupation numbers are shown in parentheses; Figure S5: Real-space representation of the HOMO (left) and LUMO (right) for the ground state of 6-acene in an OEEF of the electric field strength *F* = (a) 0.000, (b) 0.001, (c) 0.002, (d) 0.003, (e) 0.004, and (f) 0.005 a.u., calculated using spin-restricted TAO-LDA, at an isovalue of 0.02 e/Å^3^, where the orbital occupation numbers are shown in parentheses; Figure S6: Real-space representation of the HOMO (left) and LUMO (right) for the ground state of 7-acene in an OEEF of the electric field strength *F* = (a) 0.000, (b) 0.001, (c) 0.002, (d) 0.003, (e) 0.004, and (f) 0.005 a.u., calculated using spin-restricted TAO-LDA, at an isovalue of 0.02 e/Å^3^, where the orbital occupation numbers are shown in parentheses; Figure S7: Real-space representation of the HOMO (left) and LUMO (right) for the ground state of 8-acene in an OEEF of the electric field strength *F* = (a) 0.000, (b) 0.001, (c) 0.002, (d) 0.003, (e) 0.004, and (f) 0.005 a.u., calculated using spin-restricted TAO-LDA, at an isovalue of 0.02 e/Å^3^, where the orbital occupation numbers are shown in parentheses; Figure S8: Real-space representation of the HOMO (left) and LUMO (right) for the ground state of 9-acene in an OEEF of the electric field strength *F* = (a) 0.000, (b) 0.001, (c) 0.002, (d) 0.003, (e) 0.004, and (f) 0.005 a.u., calculated using spin-restricted TAO-LDA, at an isovalue of 0.02 e/Å^3^, where the orbital occupation numbers are shown in parentheses; Figure S9: Real-space representation of the HOMO (left) and LUMO (right) for the ground state of 10-acene in an OEEF of the electric field strength *F* = (a) 0.000, (b) 0.001, (c) 0.002, (d) 0.003, (e) 0.004, and (f) 0.005 a.u., calculated using spin-restricted TAO-LDA, at an isovalue of 0.02 e/Å^3^, where the orbital occupation numbers are shown in parentheses; Table S1: Singlet-triplet energy gap $E_{\mathrm{ST}}$ (in kcal/mol) of *n*-acene in an OEEF of the electric field strength *F* = 0.000, 0.001, 0.002, …, and 0.005 a.u., calculated using spin-unrestricted TAO-LDA; Table S2: Vertical ionization potential ${\mathrm{IP}}_{v}$ (in eV) for the ground state of *n*-acene in an OEEF of the electric field strength *F* = 0.000, 0.001, 0.002, …, and 0.005 a.u., calculated using spin-unrestricted TAO-LDA; Table S3: Vertical electron affinity ${\mathrm{EA}}_{v}$ (in eV) for the ground state of *n*-acene in an OEEF of the electric field strength *F* = 0.000, 0.001, 0.002, …, and 0.005 a.u., calculated using spin-unrestricted TAO-LDA; Table S4: Fundamental gap $E_{g}$ (in eV) for the ground state of *n*-acene in an OEEF of the electric field strength *F* = 0.000, 0.001, 0.002, …, and 0.005 a.u., calculated using spin-unrestricted TAO-LDA; Table S5: Symmetrized von Neumann entropy $S_{\mathrm{vN}}$ for the ground state of *n*-acene in an OEEF of the electric field strength *F* = 0.000, 0.001, 0.002, …, and 0.005 a.u., calculated using spin-unrestricted TAO-LDA.

## References

[1] Novoselov K.S., Geim A.K., Morozov S.V., Jiang D.E., Zhang Y., Dubonos S.V., Grigorieva I.V., Firsov A.A. (2004). Electric field effect in atomically thin carbon films. Science.

[2] Novoselov K.S., Geim A.K., Morozov S.V., Jiang D., Katsnelson M.I., Grigorieva I.V., Dubonos S.V., Firsov A.A. (2005). Two-dimensional gas of massless Dirac fermions in graphene. Nature.

[3] Zhang Y., Tan Y., Stormer H.L., Kim P. (2005). Experimental observation of the quantum Hall effect and Berry’s phase in graphene. Nature.

[4] Geim A.K., Novoselov K.S. (2007). The rise of graphene. Nat. Mater..

[5] Geim A.K. (2009). Graphene: Status and prospects. Science.

[6] Madurani K.A., Suprapto S., Machrita N.I., Bahar S.L., Illiya W., Kurniawan F. (2020). Progress in graphene synthesis and its application: History, challenge and the future outlook for research and industry. ECS J. Solid State Sci. Technol..

[7] Son Y.W., Cohen M.L., Louie S.G. (2006). Energy gaps in graphene nanoribbons. Phys. Rev. Lett..

[8] Han M.Y., Özyilmaz B., Zhang Y., Kim P. (2007). Energy band-gap engineering of graphene nanoribbons. Phys. Rev. Lett..

[9] Owens F.J. (2008). Electronic and magnetic properties of armchair and zigzag graphene nanoribbons. J. Chem. Phys..

[10] Lee H., Ihm J., Cohen M.L., Louie S.G. (2010). Calcium-decorated graphene-based nanostructures for hydrogen storage. Nano Lett..

[11] Kimouche A., Ervasti M.M., Drost R., Halonen S., Harju A., Joensuu P.M., Sainio J., Liljeroth P. (2015). Ultra-narrow metallic armchair graphene nanoribbons. Nat. Commun..

[12] Houtsma R.K., de la Rie J., Stöhr M. (2021). Atomically precise graphene nanoribbons: Interplay of structural and electronic properties. Chem. Soc. Rev..

[13] Wang H., Wang H.S., Ma C., Chen L., Jiang C., Chen C., Xie X., Li A.-P., Wang X. (2021). Graphene nanoribbons for quantum electronics. Nat. Rev. Phys..

[14] Saraswat V., Jacobberger R.M., Arnold M.S. (2021). Materials science challenges to graphene nanoribbon electronics. ACS Nano.

[15] Luo H., Yu G. (2022). Preparation, bandgap engineering, and performance control of graphene nanoribbons. Chem. Mater..

[16] Gu Y., Qiu Z., Müllen K. (2022). Nanographenes and graphene nanoribbons as multitalents of present and future materials science. J. Am. Chem. Soc..

[17] Friedrich N., Menchón R.E., Pozo I., Hieulle J., Vegliante A., Li J., Sánchez-Portal D., Peña D., Garcia-Lekue A., Pascual J.I. (2022). Addressing electron spins embedded in metallic graphene nanoribbons. ACS Nano.

[18] Jiang S., Neuman T., Boeglin A., Scheurer F., Schull G. (2023). Topologically localized excitons in single graphene nanoribbons. Science.

[19] Kumar S., Pratap S., Kumar V., Mishra R.K., Gwag J.S., Chakraborty B. (2023). Electronic, transport, magnetic, and optical properties of graphene nanoribbons and their optical sensing applications: A comprehensive review. Luminescence.

[20] Aragonés C., Haworth N.L., Darwish N., Ciampi S., Mannix E.J., Wallace G.G., Diez-Perez I., Coote M.L. (2016). Electrostatic catalysis of a Diels-Alder reaction. Nature.

[21] Shaik S., Mandal D., Ramanan R. (2016). Oriented electric fields as future smart reagents in chemistry. Nat. Chem..

[22] Zhang L., Laborda E., Darwish N., Noble B.B., Tyrell J.H., Pluczyk S., Le Brun A.P., Wallace G.G., Gonzalez J., Coote M.L. (2018). Electrochemical and electrostatic cleavage of alkoxyamines. J. Am. Chem. Soc..

[23] Wang Z., Danovich D., Ramanan R., Shaik S. (2018). Oriented-external electric fields create absolute enantioselectivity in Diels-Alder reactions: Importance of the molecular dipole moment. J. Am. Chem. Soc..

[24] Joy J., Stuyver T., Shaik S. (2020). Oriented external electric fields and ionic additives elicit catalysis and mechanistic crossover in oxidative addition reactions. J. Am. Chem. Soc..

[25] Shaik S., Danovich D., Joy J., Wang Z., Stuyver T. (2020). Electric-field mediated chemistry: Uncovering and exploiting the potential of (oriented) electric fields to exert chemical catalysis and reaction control. J. Am. Chem. Soc..

[26] Zhao F., Cao T., Louie S.G. (2021). Topological phases in graphene nanoribbons tuned by electric fields. Phys. Rev. Lett..

[27] Cunha L.A., Lee J., Diptarka H., McCurdy C.W., Head-Gordon M. (2021). Exploring spin symmetry-breaking effects for static field ionization of atoms: Is there an analog to the Coulson-Fischer point in bond dissociation?. J. Chem. Phys..

[28] Yu S., Vermeeren P., Hamlin T.A., Bickelhaupt F.M. (2021). How oriented external electric fields modulate reactivity. Chem. Eur. J..

[29] Wu J., Long T., Wang H., Liang J.-X., Zhu C. (2022). Oriented external electric fields regurating the reaction mechanism of CH_4_ oxidation catalyzed by Fe(IV)-Oxo-corrolazine: Insight from density functional calculations. Front. Chem..

[30] Scheele T., Neudecker T. (2023). Investigating the accuracy of density functional methods for molecules in electric fields. J. Chem. Phys..

[31] Scheele T., Neudecker T. (2023). Using oriented external electric fields to manipulate rupture forces of mechanophores. Phys. Chem. Chem. Phys..

[32] Hachmann J., Dorando J.J., Aviles M., Chan G.K.L. (2007). The radical character of the acenes: A density matrix renormalization group study. J. Chem. Phys..

[33] Chai J.-D. (2012). Density functional theory with fractional orbital occupations. J. Chem. Phys..

[34] Mizukami W., Kurashige Y., Yanai T. (2013). More *π* electrons make a difference: Emergence of many radicals on graphene nanoribbons studied by ab initio DMRG theory. J. Chem. Theory Comput..

[35] Rivero P., Jiménez-Hoyos C.A., Scuseria G.E. (2013). Entanglement and polyradical nature of polycyclic aromatic hydrocarbons predicted by projected Hartree-Fock theory. J. Phys. Chem. B.

[36] Chai J.-D. (2014). Thermally-assisted-occupation density functional theory with generalized-gradient approximations. J. Chem. Phys..

[37] Wu C.-S., Chai J.-D. (2015). Electronic properties of zigzag graphene nanoribbons studied by TAO-DFT. J. Chem. Theory Comput..

[38] Fosso-Tande J., Nguyen T.-S., Gidofalvi G., DePrince A.E. (2016). Large-scale variational two-electron reduced-density-matrix-driven complete active space self-consistent field methods. J. Chem. Theory Comput..

[39] Chai J.-D. (2017). Role of exact exchange in thermally-assisted-occupation density functional theory: A proposal of new hybrid schemes. J. Chem. Phys..

[40] Chen B.-J., Chai J.-D. (2022). TAO-DFT fictitious temperature made simple. RSC Adv..

[41] Dai Y., Sancho-García J.-C., Negri F. (2023). Impact of di- and poly-radical characters on the relative energy of the doubly excited and *L*_a_ states of linear acenes and cyclacenes. Chemistry.

[42] Hohenberg P., Kohn W. (1964). Inhomogeneous electron gas. Phys. Rev..

[43] Kohn W., Sham L.J. (1965). Self-consistent equations including exchange and correlation effects. Phys. Rev..

[44] Dirac P.A.M. (1930). Note on exchange phenomena in the Thomas atom. Proc. Camb. Philos. Soc..

[45] Perdew J.P., Wang Y. (1992). Accurate and simple analytic representation of the electron-gas correlation energy. Phys. Rev. B.

[46] Perdew J.P., Burke K., Ernzerhof M. (1996). Generalized gradient approximation made simple. Phys. Rev. Lett..

[47] Becke A.D. (1993). A new mixing of Hartree-Fock and local density-functional theories. J. Chem. Phys..

[48] Becke A.D. (1993). Density-functional thermochemistry. III. The role of exact exchange. J. Chem. Phys..

[49] Stephens P.J., Devlin F.J., Chabalowski C.F., Frisch M.J. (1994). Ab initio calculation of vibrational absorption and circular dichroism spectra using density functional force fields. J. Phys. Chem..

[50] Kümmel S., Kronik L. (2008). Orbital-dependent density functionals: Theory and applications. Rev. Mod. Phys..

[51] Cohen A.J., Mori-Sánchez P., Yang W. (2008). Insights into current limitations of density functional theory. Science.

[52] Cohen A.J., Mori-Sánchez P., Yang W. (2012). Challenges for density functional theory. Chem. Rev..

[53] Teale A.M., Helgaker T., Savin A., Adamo C., Aradi B., Arbuznikov A.V., Ayers P.W., Baerends E.J., Barone V., Calaminici P. (2022). DFT Exchange: Sharing perspectives on the workhorse of quantum chemistry and materials science. Phys. Chem. Chem. Phys..

[54] Andersson K., Malmqvist P.-Å., Roos B.O. (1992). Second-order perturbation theory with a complete active space self-consistent field reference function. J. Chem. Phys..

[55] Gidofalvi G., Mazziotti D.A. (2008). Active-space two-electron reduced-density-matrix method: Complete active-space calculations without diagonalization of the *N*-electron hamiltonian. J. Chem. Phys..

[56] Gryn’ova G., Coote M.L., Corminboeuf C. (2015). Theory and practice of uncommon molecular electronic configurations. WIREs Comput. Mol. Sci..

[57] Goli V.D.P., Prodhan S., Mazumdar S., Ramasesha S. (2016). Correlated electronic properties of some graphene nanoribbons: A DMRG study. Phys. Rev. B.

[58] Hagymási I., Legeza Ö. (2016). Entanglement, excitations, and correlation effects in narrow zigzag graphene nanoribbons. Phys. Rev. B.

[59] Piris M. (2017). Global method for electron correlation. Phys. Rev. Lett..

[60] van Meer R., Gritsenko O.V., Baerends E.J. (2018). A non-JKL density matrix functional for intergeminal correlation between closed-shell geminals from analysis of natural orbital configuration interaction expansions. J. Chem. Phys..

[61] Wang Y.-Y., Chai J.-D. (2024). Spin symmetry in thermally-assisted-occupation density-functional theory. Phys. Rev. A.

[62] Xuan F., Chai J.-D., Su H. (2019). Local density approximation for the short-range exchange free energy functional. ACS Omega.

[63] Grimme S. (2006). Semiempirical GGA-type density functional constructed with a long-range dispersion correction. J. Comput. Chem..

[64] Grimme S., Hansen A., Brandenburg J.G., Bannwarth C. (2016). Dispersion-corrected mean-field electronic structure methods. Chem. Rev..

[65] Seenithurai S., Chai J.-D. (2016). Effect of Li adsorption on the electronic and hydrogen storage properties of acenes: A dispersion-corrected TAO-DFT study. Sci. Rep..

[66] Lin C.-Y., Hui K., Chung J.-H., Chai J.-D. (2017). Self-consistent determination of the fictitious temperature in thermally-assisted-occupation density functional theory. RSC Adv..

[67] Mermin N.D. (1965). Thermal properties of the inhomogeneous electron gas. Phys. Rev..

[68] Tsai H.-Y., Chai J.-D. (2023). Real-time extension of TAO-DFT. Molecules.

[69] Seenithurai S., Chai J.-D. (2023). TAO-DFT with the polarizable continuum model. Nanomaterials.

[70] Li S., Chai J.-D. (2020). TAO-DFT-based ab initio molecular dynamics. Front. Chem..

[71] Yeh C.-N., Chai J.-D. (2016). Role of Kekulé and non-Kekulé structures in the radical character of alternant polycyclic aromatic hydrocarbons: A TAO-DFT study. Sci. Rep..

[72] Yeh C.-N., Wu C., Su H., Chai J.-D. (2018). Electronic properties of the coronene series from thermally-assisted-occupation density functional theory. RSC Adv..

[73] Tönshoff C., Bettinger H.F. (2021). Pushing the limits of acene chemistry: The recent surge of large acenes. Chem. Eur. J..

[74] Gupta D., Omont A., Bettinger H.F. (2021). Energetics of formation of cyclacenes from 2,3-didehydroacenes and implications for astrochemistry. Chem. Eur. J..

[75] Nieman R., Carvalho J.R., Jayee B., Hansen A., Aquino A.J., Kertesz M., Lischka H. (2023). Polyradical character assessment using multireference calculations and comparison with density-functional derived fractional occupation number weighted density analysis. Phys. Chem. Chem. Phys..

[76] Somani A., Gupta D., Bettinger H.F. (2024). Computational studies of dimerization of [*n*]-cyclacenes. J. Phys. Chem. A.

[77] Hanson-Heine M.W.D. (2020). Static correlation in vibrational frequencies studied using thermally-assisted-occupation density functional theory. Chem. Phys. Lett..

[78] Hanson-Heine M.W.D. (2022). Static electron correlation in anharmonic molecular vibrations: A hybrid TAO-DFT study. J. Phys. Chem. A.

[79] Shao Y., Gan Z., Epifanovsky E., Gilbert A.T., Wormit M., Kussmann J., Lange A.W., Behn A., Deng J., Feng X. (2015). Advances in molecular quantum chemistry contained in the Q-Chem 4 program package. Mol. Phys..

[80] Su Y., Wang X., Wang L., Zhang Z., Wang X., Song Y., Power P.P. (2016). Thermally controlling the singlet–triplet energy gap of a diradical in the solid state. Chem. Sci..

[81] Yu L., Wu Z., Xie G., Zhong C., Zhu Z., Cong H., Ma D., Yang C. (2016). Achieving a balance between small singlet-triplet energy splitting and high fluorescence radiative rate in a quinoxaline-based orange-red thermally activated delayed fluorescence emitter. Chem. Commun..

[82] Smith M.B., Michl J. (2010). Singlet fission. Chem. Rev..

[83] Zhou J., Liu Q., Feng W., Sun Y., Li F. (2015). Upconversion luminescent materials: Advances and applications. Chem. Rev..

[84] Romero N.A., Nicewicz D.A. (2016). Organic photoredox catalysis. Chem. Rev..

[85] Xia J., Sanders S.N., Cheng W., Low J.Z., Liu J., Campos L.M., Sun T. (2017). Singlet fission: Progress and prospects in solar cells. Adv. Mater..

[86] Löwdin P.-O., Shull H. (1956). Natural orbitals in the quantum theory of two-electron systems. Phys. Rev..

