# Supplementary material for: Effect of Oriented External Electric Fields on the Electronic Properties of Linear Acenes: A Thermally Assisted Occupation DFT Study
Source: Molecules. 2024 Sep 6;29(17):4245. doi: 10.3390/molecules29174245 (PMC11396984; doi:10.3390/molecules29174245)
Supplement: Supplementary file 1 [file molecules-29-04245-s001.zip › molecules-3188754-supplementary.pdf]

Supplementary Information to: Effect of Oriented External Electric Fields on the Electronic Properties of Linear Acenes: A Thermally Assisted Occupation DFT Study

Chi-Yu Chen[1] and Jeng-Da Chai[1,2,3,*]

[1] *Department of Physics, National Taiwan University, Taipei 10617, Taiwan*

[2] *Center for Theoretical Physics and Center for Quantum Science and Engineering, National Taiwan University, Taipei 10617, Taiwan*

[3] *Physics Division, National Center for Theoretical Sciences, Taipei 10617, Taiwan*

[*] Author to whom correspondence should be addressed. Electronic mail: jdchai@phys.ntu.edu.tw

FIGURES

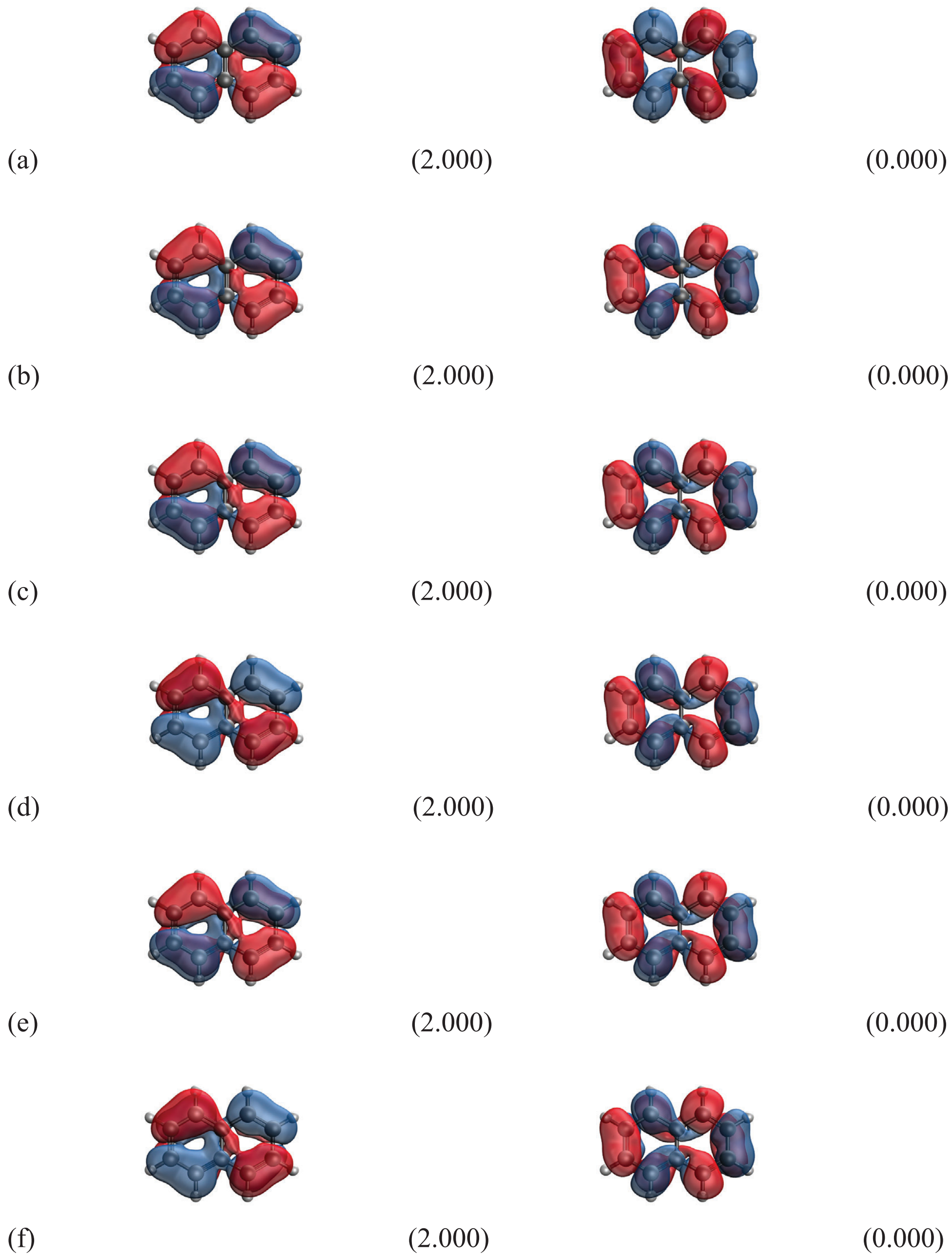

FIG. S1. Real-space representation of the HOMO (left) and LUMO (right) for the ground state of 2-acene in an OEEF of the electric field strength $F =$ (a) 0.000, (b) 0.001, (c) 0.002, (d) 0.003, (e) 0.004, and (f) 0.005 a.u., calculated using spin-restricted TAO-LDA, at an isovalue of 0.02 e/Å^3, where the orbital occupation numbers are shown in parentheses.

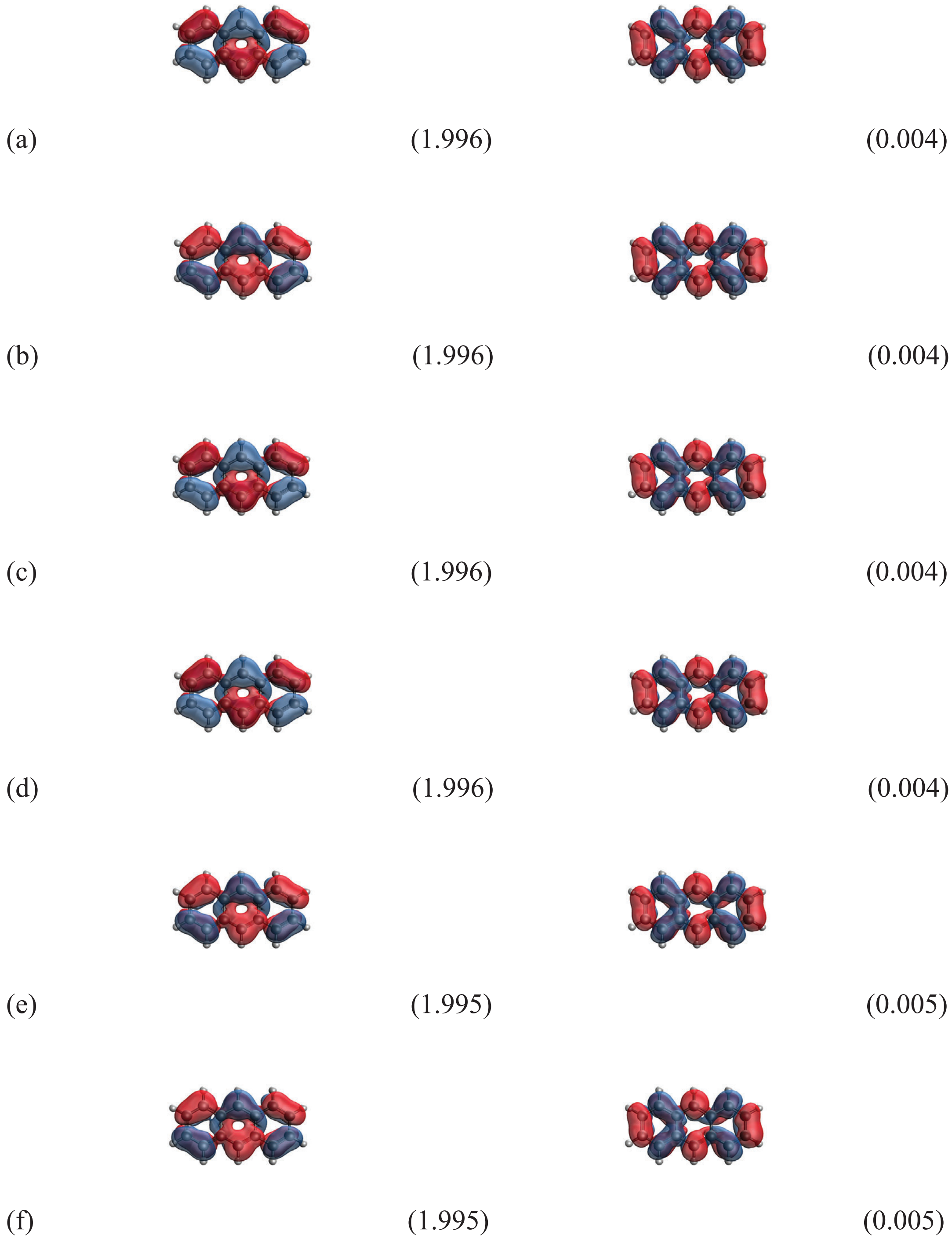

FIG. S2. Real-space representation of the HOMO (left) and LUMO (right) for the ground state of 3-acene in an OEEF of the electric field strength $F =$ (a) 0.000, (b) 0.001, (c) 0.002, (d) 0.003, (e) 0.004, and (f) 0.005 a.u., calculated using spin-restricted TAO-LDA, at an isovalue of 0.02 e/Å^3, where the orbital occupation numbers are shown in parentheses.

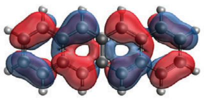 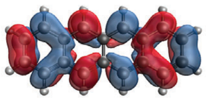

(a) (1.972) (0.028)

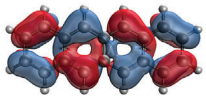 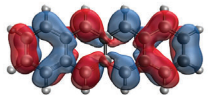

(b) (1.972) (0.028)

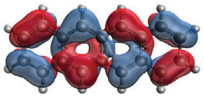 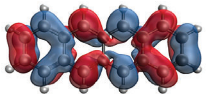

(c) (1.971) (0.029)

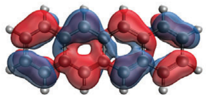 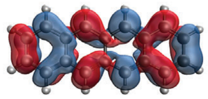

(d) (1.970) (0.030)

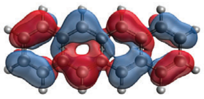 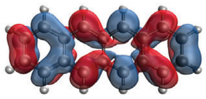

(e) (1.968) (0.033)

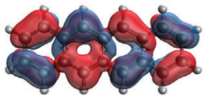 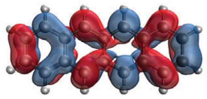

(f) (1.965) (0.035)

FIG. S3. Real-space representation of the HOMO (left) and LUMO (right) for the ground state of 4-acene in an OEEF of the electric field strength $F =$ (a) 0.000, (b) 0.001, (c) 0.002, (d) 0.003, (e) 0.004, and (f) 0.005 a.u., calculated using spin-restricted TAO-LDA, at an isovalue of 0.02 e/Å^3, where the orbital occupation numbers are shown in parentheses.

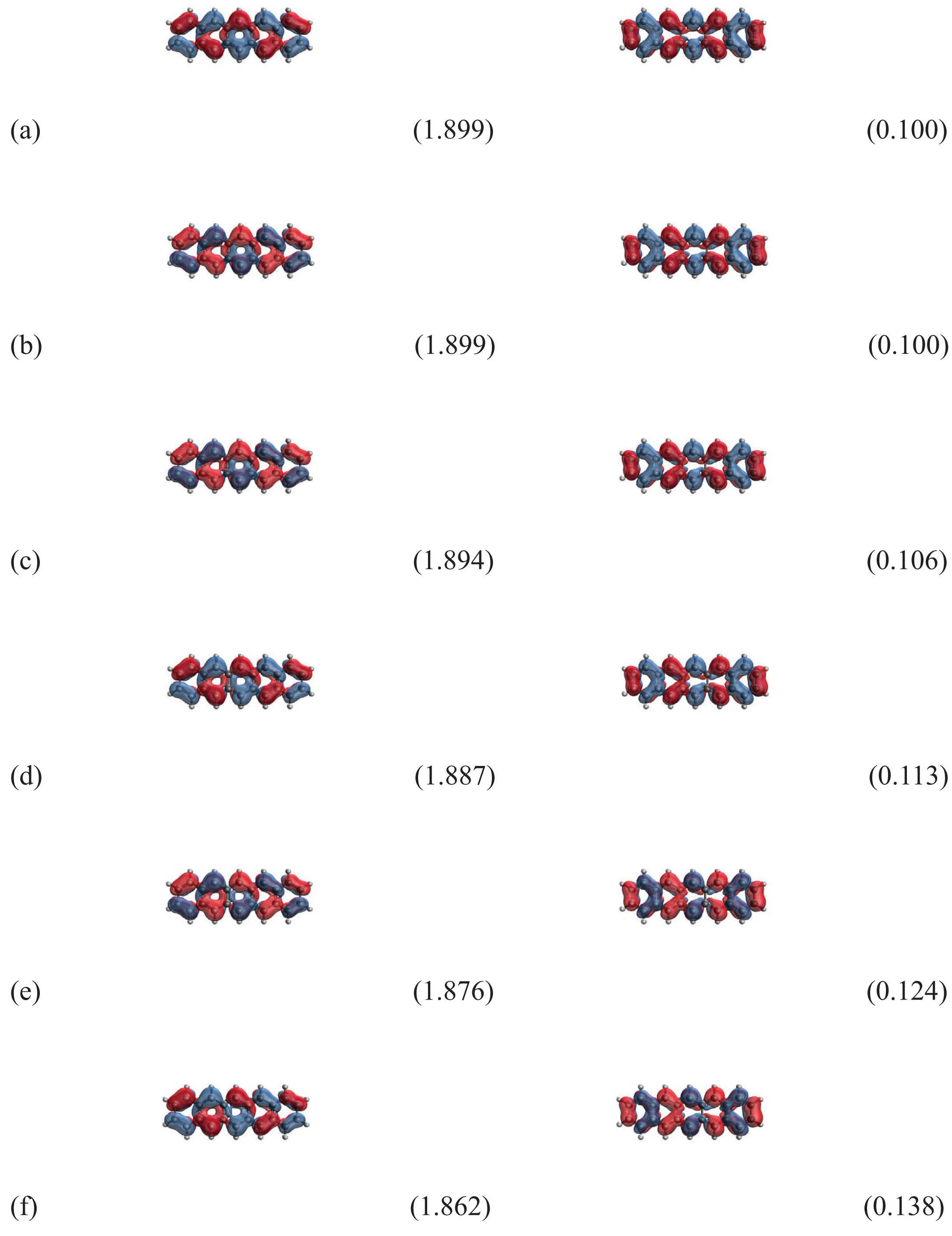

FIG. S4. Real-space representation of the HOMO (left) and LUMO (right) for the ground state of 5-acene in an OEEF of the electric field strength F = (a) 0.000, (b) 0.001, (c) 0.002, (d) 0.003, (e) 0.004, and (f) 0.005 a.u., calculated using spin-restricted TAO-LDA, at an isovalue of 0.02 e/Å^3, where the orbital occupation numbers are shown in parentheses.

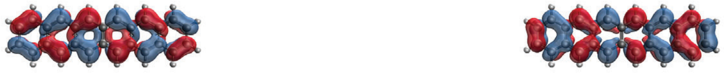

(a) (1.755) (0.242)

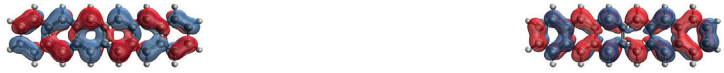

(b) (1.755) (0.242)

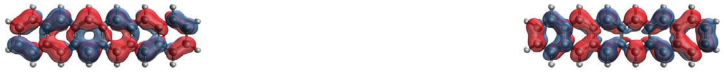

(c) (1.744) (0.257)

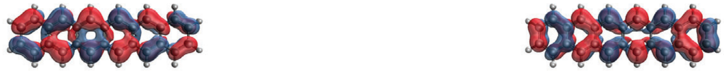

(d) (1.725) (0.276)

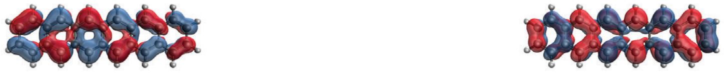

(e) (1.699) (0.302)

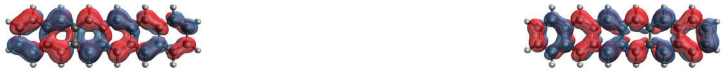

(f) (1.666) (0.335)

FIG. S5. Real-space representation of the HOMO (left) and LUMO (right) for the ground state of 6-acene in an OEEF of the electric field strength $F =$ (a) 0.000, (b) 0.001, (c) 0.002, (d) 0.003, (e) 0.004, and (f) 0.005 a.u., calculated using spin-restricted TAO-LDA, at an isovalue of 0.02 e/Å^3, where the orbital occupation numbers are shown in parentheses.

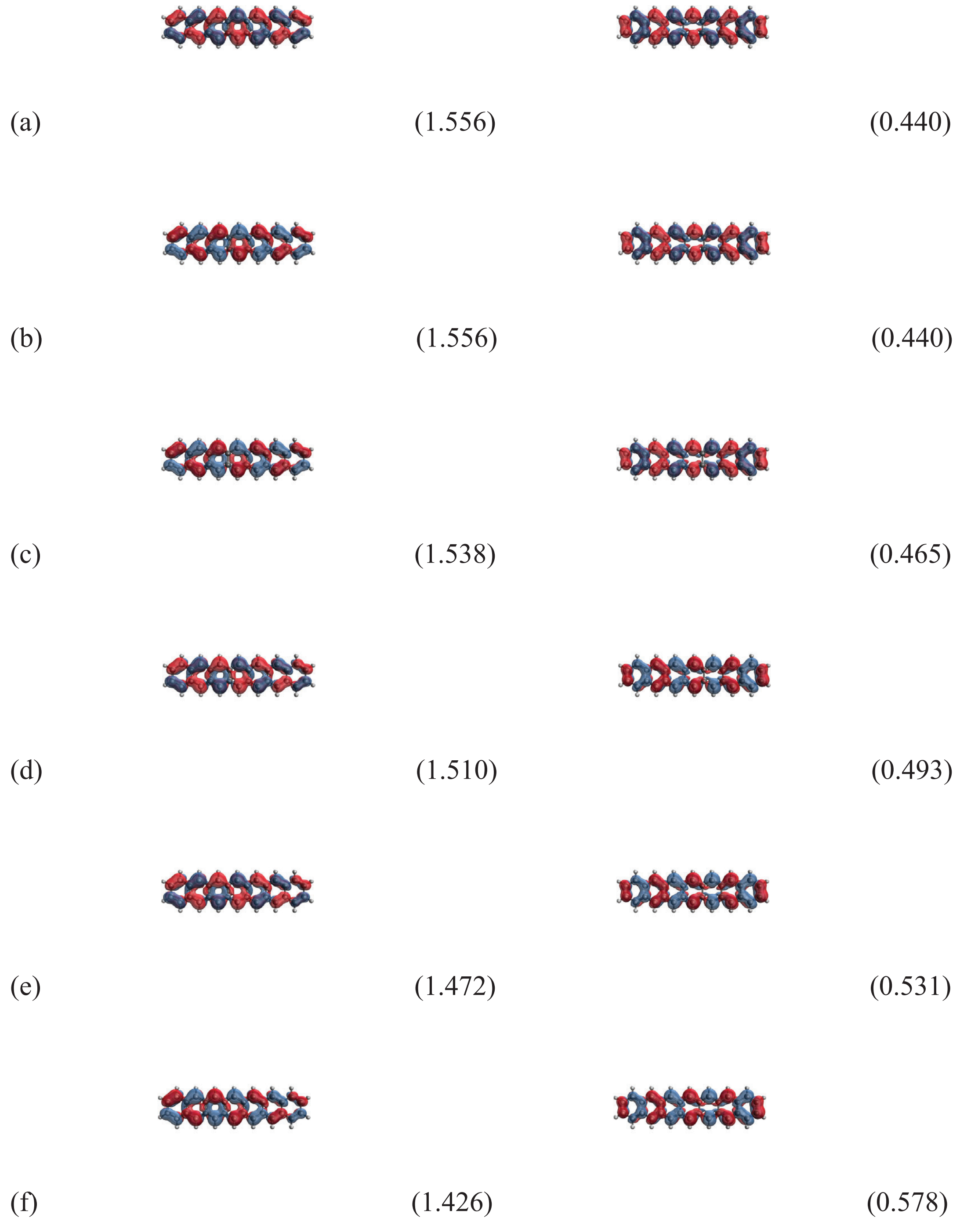

FIG. S6. Real-space representation of the HOMO (left) and LUMO (right) for the ground state of 7-acene in an OEEF of the electric field strength $F =$ (a) 0.000, (b) 0.001, (c) 0.002, (d) 0.003, (e) 0.004, and (f) 0.005 a.u., calculated using spin-restricted TAO-LDA, at an isovalue of 0.02 e/Å^3, where the orbital occupation numbers are shown in parentheses.

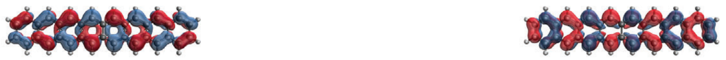

(a) (1.346) (0.653)

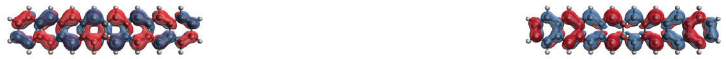

(b) (1.346) (0.653)

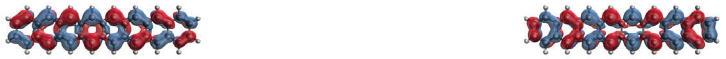

(c) (1.325) (0.682)

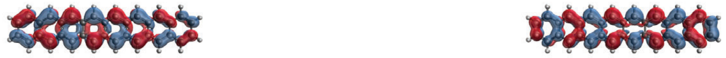

(d) (1.292) (0.715)

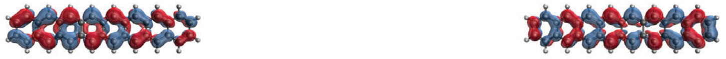

(e) (1.249) (0.758)

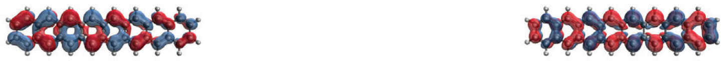

(f) (1.199) (0.809)

FIG. S7. Real-space representation of the HOMO (left) and LUMO (right) for the ground state of 8-acene in an OEEF of the electric field strength $F =$ (a) 0.000, (b) 0.001, (c) 0.002, (d) 0.003, (e) 0.004, and (f) 0.005 a.u., calculated using spin-restricted TAO-LDA, at an isovalue of 0.02 e/Å^3, where the orbital occupation numbers are shown in parentheses.

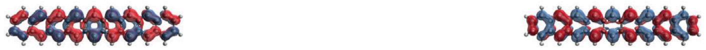

(a) (1.161) (0.843)

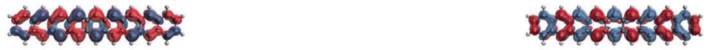

(b) (1.161) (0.843)

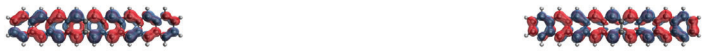

(c) (1.139) (0.874)

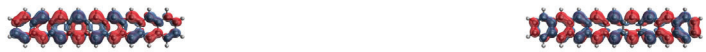

(d) (1.105) (0.908)

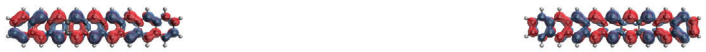

(e) (1.062) (0.953)

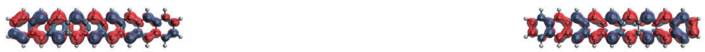

(f) (1.011) (1.005)

FIG. S8. Real-space representation of the HOMO (left) and LUMO (right) for the ground state of 9-acene in an OEEF of the electric field strength F = (a) 0.000, (b) 0.001, (c) 0.002, (d) 0.003, (e) 0.004, and (f) 0.005 a.u., calculated using spin-restricted TAO-LDA, at an isovalue of 0.02 e/Å^3, where the orbital occupation numbers are shown in parentheses.

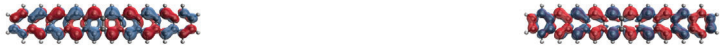

(a) (1.012) (1.000)

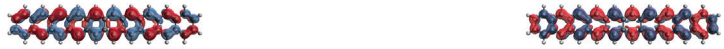

(b) (1.012) (1.000)

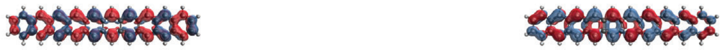

(c) (1.030) (0.991)

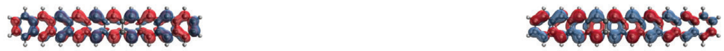

(d) (1.065) (0.957)

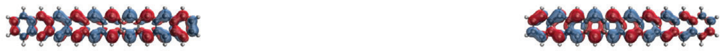

(e) (1.109) (0.915)

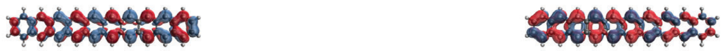

(f) (1.161) (0.865)

FIG. S9. Real-space representation of the HOMO (left) and LUMO (right) for the ground state of 10-acene in an OEEF of the electric field strength $F =$ (a) 0.000, (b) 0.001, (c) 0.002, (d) 0.003, (e) 0.004, and (f) 0.005 a.u., calculated using spin-restricted TAO-LDA, at an isovalue of 0.02 e/Å^3, where the orbital occupation numbers are shown in parentheses.

TABLES

TABLE S1. Singlet-triplet energy gap E_{ST} (in kcal/mol) of n-acene in an OEEF of the electric field strength $F = 0.000, 0.001, 0.002, ...,$ and 0.005 a.u., calculated using spin-unrestricted TAO-LDA.

n	$F = 0.000$	$F = 0.001$	$F = 0.002$	$F = 0.003$	$F = 0.004$	$F = 0.005$
2	64.77	64.77	64.75	64.72	64.67	64.62
3	43.22	43.20	43.15	43.06	42.95	42.79
4	29.01	28.98	28.89	28.73	28.51	28.22
5	19.60	19.55	19.41	19.18	18.87	18.48
6	13.55	13.50	13.35	13.11	12.80	12.42
7	9.91	9.87	9.75	9.57	9.33	9.07
8	7.85	7.82	7.74	7.63	7.50	7.37
9	6.66	6.65	6.61	6.55	6.50	6.46
10	5.91	5.90	5.87	5.85	5.83	5.83

TABLE S2. Vertical ionization potential IP_v (in eV) for the ground state of n-acene in an OEEF of the electric field strength $F = 0.000, 0.001, 0.002, ...,$ and 0.005 a.u., calculated using spin-unrestricted TAO-LDA.

n	$F = 0.000$	$F = 0.001$	$F = 0.002$	$F = 0.003$	$F = 0.004$	$F = 0.005$
2	7.81	7.81	7.81	7.81	7.80	7.80
3	7.00	7.07	7.13	7.19	7.25	7.31
4	6.46	6.46	6.45	6.45	6.44	6.43
5	6.07	6.13	6.18	6.24	6.29	6.34
6	5.79	5.78	5.78	5.77	5.75	5.74
7	5.59	5.65	5.70	5.76	5.81	5.86
8	5.44	5.44	5.43	5.43	5.42	5.41
9	5.33	5.39	5.45	5.50	5.56	5.61
10	5.23	5.23	5.23	5.22	5.21	5.21

TABLE S3. Vertical electron affinity EA_v (in eV) for the ground state of n-acene in an OEEF of the electric field strength F = 0.000, 0.001, 0.002, ..., and 0.005 a.u., calculated using spin-unrestricted TAO-LDA.

n	$F = 0.000$	$F = 0.001$	$F = 0.002$	$F = 0.003$	$F = 0.004$	$F = 0.005$
2	-0.62	-0.62	-0.62	-0.62	-0.62	-0.61
3	0.28	0.34	0.41	0.48	0.54	0.62
4	0.90	0.90	0.90	0.91	0.92	0.94
5	1.34	1.40	1.47	1.55	1.63	1.71
6	1.66	1.66	1.67	1.68	1.70	1.72
7	1.89	1.96	2.03	2.10	2.18	2.26
8	2.06	2.06	2.07	2.08	2.10	2.11
9	2.19	2.26	2.33	2.40	2.48	2.55
10	2.30	2.30	2.31	2.32	2.34	2.35

TABLE S4. Fundamental gap E_g (in eV) for the ground state of n-acene in an OEEF of the electric field strength F = 0.000, 0.001, 0.002, ..., and 0.005 a.u., calculated using spin-unrestricted TAO-LDA.

n	$F = 0.000$	$F = 0.001$	$F = 0.002$	$F = 0.003$	$F = 0.004$	$F = 0.005$
2	8.43	8.43	8.43	8.43	8.42	8.42
3	6.73	6.72	6.72	6.71	6.70	6.69
4	5.56	5.56	5.55	5.54	5.52	5.49
5	4.73	4.72	4.71	4.69	4.66	4.63
6	4.13	4.12	4.11	4.08	4.05	4.02
7	3.69	3.69	3.68	3.66	3.63	3.60
8	3.38	3.37	3.36	3.34	3.32	3.30
9	3.13	3.13	3.12	3.10	3.08	3.06
10	2.93	2.93	2.91	2.90	2.88	2.86

TABLE S5. Symmetrized von Neumann entropy S_{vN} for the ground state of n-acene in an OEEF of the electric field strength F = 0.000, 0.001, 0.002, ..., and 0.005 a.u., calculated using spin-unrestricted TAO-LDA.

n	$F = 0.000$	$F = 0.001$	$F = 0.002$	$F = 0.003$	$F = 0.004$	$F = 0.005$
2	0.00	0.00	0.00	0.00	0.00	0.00
3	0.03	0.03	0.03	0.03	0.03	0.03
4	0.15	0.15	0.15	0.16	0.17	0.18
5	0.40	0.40	0.42	0.44	0.47	0.50
6	0.75	0.75	0.78	0.81	0.86	0.91
7	1.08	1.09	1.11	1.15	1.19	1.24
8	1.34	1.34	1.36	1.39	1.42	1.45
9	1.52	1.52	1.54	1.55	1.58	1.60
10	1.67	1.67	1.68	1.70	1.72	1.74
